# FunFOLDQA: A Quality Assessment Tool for Protein-Ligand Binding Site Residue Predictions

**DOI:** 10.1371/journal.pone.0038219

**Published:** 2012-05-30

**Authors:** Daniel B. Roche, Maria T. Buenavista, Liam J. McGuffin

**Affiliations:** 1 School of Biological Sciences, University of Reading, Reading, Berkshire, United Kingdom; 2 Biocomputing Section, Medical Research Council Harwell, Harwell Oxford, Oxfordshire, United Kingdom; 3 Beamline B23, Diamond Light Source, Didcot, Oxfordshire, United Kingdom; University of Alberta, Canada

## Abstract

The estimation of prediction quality is important because without quality measures, it is difficult to determine the usefulness of a prediction. Currently, methods for ligand binding site residue predictions are assessed in the function prediction category of the biennial Critical Assessment of Techniques for Protein Structure Prediction (CASP) experiment, utilizing the Matthews Correlation Coefficient (MCC) and Binding-site Distance Test (BDT) metrics. However, the assessment of ligand binding site predictions using such metrics requires the availability of solved structures with bound ligands. Thus, we have developed a ligand binding site quality assessment tool, FunFOLDQA, which utilizes protein feature analysis to predict ligand binding site quality prior to the experimental solution of the protein structures and their ligand interactions. The FunFOLDQA feature scores were combined using: simple linear combinations, multiple linear regression and a neural network. The neural network produced significantly better results for correlations to both the MCC and BDT scores, according to Kendall’s *τ*, Spearman’s *ρ* and Pearson’s *r* correlation coefficients, when tested on both the CASP8 and CASP9 datasets. The neural network also produced the largest Area Under the Curve score (AUC) when Receiver Operator Characteristic (ROC) analysis was undertaken for the CASP8 dataset. Furthermore, the FunFOLDQA algorithm incorporating the neural network, is shown to add value to FunFOLD, when both methods are employed in combination. This results in a statistically significant improvement over all of the best server methods, the FunFOLD method (6.43%), and one of the top manual groups (FN293) tested on the CASP8 dataset. The FunFOLDQA method was also found to be competitive with the top server methods when tested on the CASP9 dataset. To the best of our knowledge, FunFOLDQA is the first attempt to develop a method that can be used to assess ligand binding site prediction quality, in the absence of experimental data.

## Introduction

Proteins are essential molecules in all living organisms and are involved in virtually all cellular processes, including; transportation within and between cells, energy generation, catalysis, signalling, defence and maintaining the structural integrity of cells. Determining a protein’s ligand binding site location and potential interacting residues is important for; functional determination, mutagenesis studies, ligand binding site specificity and *de novo drug* design [1], [2], [3], [4], [5].

The development of numerous protein ligand binding site prediction methods has been driven by the recent inclusion of the function prediction category in CASP [6]. Ligand binding site prediction methods are subdivided into two broad groupings: sequence-based methods and structure based-methods [7]. The sequence based methods utilize sequence conservations of structurally or functionally important residues, these methods include firestar (CASP9 – group FN315) [8], [9], WSsas [10], FRcons [11], ConFunc (CASP8 - FN437) [12], ConSurf [13], FPSDP (CASP8 - FN242) [14], INTREPID [15] and ss-TEA [16]. Structure based methods can be further separated into geometric methods (FINDSITE [17] and Surflex-PSIM [18]), energetic methods (SITEHOUND [19]) and miscellaneous methods, which utilize knowledge from homology modelling (FunFOLD – CASP9 FN425 [4], 3DLigandSite –CASP9 FN017, FN057, FN072 and FN415 [20] and I-TASSER_FUNCTION – CASP9 FN339 [21]), surface accessibility (LIGSITE^CSC^ [22]) and physiochemical properties (SCREEN [23]).

The top function prediction methods in CASP8 were the manual methods by the Lee group [7] and the Sternberg group [24]. Both groups used the superposition of structurally similar templates containing biologically relevant ligands, onto protein models, in order to determine the location of the ligand binding site and the residues involved in binding [7], [24]. Since CASP8 the Sternberg group developed a web server for their algorithm 3DLigandSite [20] (http://www.sbg.bio.ic.ac.uk/3dligandsite/).

In CASP9 many of the top performing servers, with the exception of firestar [8], [9], converged on the similar concept of structural superpositions of models to templates for predicting ligand binding site locations [25]. For example, of the top 10 performing methods in CASP9, the FunFOLD method (McGuffin) [4], the Lee group [7], the Sternberg group [24] and the Zhang group all implemented methods based on this idea. In addition to carrying out structural superpositions of templates containing biologically relevant ligands onto the model, the Zhang group (I-TASSER_FUNCTION [21]), additionally carried out local superpositions of predicted binding sites of the templates to the model, which was thought to have helped to increase their accuracy marginally in relation to other groups.

In CASP8, the function prediction category was assessed using the Matthews Correlation Coefficient (MCC) [1]. The MCC is a statistical metric for the comparison of the predicted ligand binding site residues to the observed ligand binding site residues, by comparing the number of residues assigned as true positive, false positive, true negative and false negative, resulting in a score between −1 and 1. A perfect prediction receives a score of 1, whereas a random prediction receives a score close to 0 [3]. The MCC score penalizes both over and under predictions making it a good assessment metric, but the observed binding site residues need to be clearly defined. However, it is difficult to conclusively define, which residues will bind to a ligand, considering the inherent flexibility of proteins and ligands, and proteins may bind to multiple ligands in the same binding site.

In CASP9 both the Matthews Correlation Coefficient (MCC) and the Binding-site Distance Test (BDT) score were used in the assessment of ligand binding site residue predictions [25]. The BDT metric addressed some of the problems associated with the MCC score, while maintaining the advantages. The BDT score ranges from 0 to 1, where a score close to 0 is a random prediction and 1 is a perfect prediction. The BDT score takes into account the distance a predicted binding site residue is from an observed binding site residue, assigning a score accordingly. Binding site residues predicted to be close to the observed binding site residues receive higher scores than more distant residues [3]. Both the MCC and BDT metrics are used to analyse a prediction, *after* the experimental protein structure data is available.

In recent years Quality Assessment (QA) has gained attention to become an integral part of tertiary structure prediction [26], and here we are proposing that similar metrics should become an integral component of ligand binding site residue predictions. Protein feature analysis is incorporated into numerous QA and ligand binding site prediction tools. Cheng and co-workers built a single model QA tool, which exploits structural features, integrated into a support vector machine to predict model quality [27]. The Cheng group have since developed numerous other feature based QA tools, MULITCOM [28] a consensus-based method and most recently APOLLO [29] a single model-based assessment tool. Both MULTICOM and APOLLO integrate feature analysis for secondary structure, solvent accessibility, contact maps and beta-sheet topology [28], [29]. Several other QA methods also integrate various protein features including QMEAN and QMEANclust [30], [31], ProQ [32] and a more recent method by Kalman and Ben-Tal [33]. Several methods also incorporate protein feature analysis for the prediction of ligand binding site residues, these methods include DISCERN [5], a meta-functional signature method by Wang et al [34] and a carbohydrate-binding module, binding site residue prediction method [35].

Although it is clear that numerous algorithms have been developed to incorporate protein feature analysis for protein structure prediction, global model quality assessment and ligand binding site residue prediction, we were unable to find any methods that explicitly use protein feature analysis to assess ligand binding site prediction quality. In this paper, we describe the FunFOLDQA method, which can be used for the assessment of ligand binding site prediction quality, *prior* to the availability of experimental structural data. For experimentalists it is important to know, which predictions they can trust and use to formulate new hypotheses and plan new experiments. The availability of predicted quality scores that correlate well with observed quality scores will provide the necessary confidence measures for assessing ligand binding site residue predictions.

## Methods

### The FunFOLD Method

The FunFOLD algorithm, which has been described previously [4], uses structural superpositions of the top ranked models and related templates with bound ligands in order to identify putative contacting residues. The method uses a novel fully automated agglomerative clustering approach for both ligand identification and residue selection. The FunFOLDQA feature scores (described below) are derived from data generated by running the FunFOLD method. However, similar data are also produced by the majority of the top structure based binding site residue prediction methods.

### The FunFOLDQA Feature Scores

Initially 10 different feature dependent scores, ranging between 0 and 1 were developed; 4 binding site dependent feature scores; 3 ligand dependent feature scores and 3 structure dependent feature scores. The scores were based on several features we found to be important in determining a confident prediction from our development work for the FunFOLD algorithm and from our manual function prediction submissions for CASP9. A detailed overview of all feature dependent scores developed for the FunFOLDQA algorithm follows:

#### Binding site dependent feature scores

*BDTalign*: The BDTalign score determines the distance between equivalent residues of the model binding site and each template binding site in 3D space. In other words it is simply a measure of the structural fit between the template binding site and the model binding site. The BDTalign score is an extension of the original BDT score [3], but compares superposed binding sites of templates to models, as opposed to the BDT score, which compares a predicted and observed binding site on the same protein. The BDTalign score was calculated by considering: the list of residue numbers in the model predicted to be binding to a ligand, the list of residue numbers of each template predicted to be binding to the biologically relevant ligand (the distance cut-off for contacting atoms was 0.5 Å plus the Van der Waal radii, which is a CASP parameter) and the model and template structures. The models and templates were superposed using TM-align [36], the Euclidean distance was then calculated between each binding site residue in the model and each binding site residue in the template. The distance was then converted to an *S*-score using the standard equation:
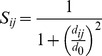
Where *S_ij_* was the *S*-score between a predicted residue in the model *i* and a binding site residue in the template *j*, *d_ij_* was the Euclidean distance between the C-alpha coordinates of residues *i* and *j* and *d*_0_ was a distance threshold (3Å – the upper range cutoff for the BDT score). The maximum *S_ij_* score, max(*S_ij_*), was then determined for each predicted residue, which was the binding site residue in the model to the closest equivalent binding site residue in the template. The Template BDTalign score, was simply the sum of the maximum *S_ij_* scores, divided by the greater value of the number of predicted residues in the model (*N_p_*) and the number of residues in the template (*N_t_*):



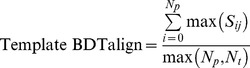
The final BDTalign score was the sum of all the Template BDTalign scores normalized by the total number of templates used in the prediction (*N_tot_*):



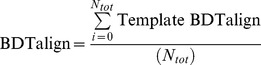



*Identity:* The Identity score determines the binding residues that are “equivalent” in 3D space in the model and the template binding site and scores them according to their amino acid identity. The Identity score was calculated, by firstly determining the closest equivalent residues in the model binding site, to residues in the template binding site, for each template, by calculating an *S*-score as in the BDTalign method using the standard equation. If equivalent residues were the same amino acid, then the Equivalent Residue score was equal to 1, if the equivalent residue amino acids were not equal, the Equivalent Residue score was 0. The Identity score for each template (Template Identity) was then calculated, which was simply the sum of the Equivalent Residue scores divided by the greater value of the number of predicted residues in the model (*N_p_*) and the number of residues in the template (*N_t_*):
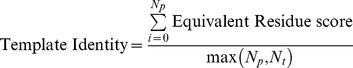
The final Identity score was the sum of all the Template Identity scores normalized by the total number of templates used in the prediction (*N_tot_*):



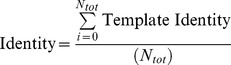



*Rescaled BLOSUM62 score:* The Rescaled BLOSUM62 score scores residues that are equivalent in 3D space, according to the BLOSUM62 scoring matrix. The BLOSUM62 matrix was chosen as it is a widely used as a default substitution matrix, in numerous sequence alignment algorithms. The Rescaled BLOSUM62 score was calculated, in a similar way to the Identity score. However, the closest equivalent residues were scored using the BLOSUM62 substitution matrix. The BLOSUM62 score for each template (Template BLOSUM62 score) was then calculated, which was simply the sum of the equivalent residue BLOSUM62 scores, plus the number of extra binding site residues either in the model or in the template (*diff*(−4)). The score of −4 for each extra residue is given to prevent biasing over-predictions or under-predictions. The score was then divided by the greater value of the number of predicted residues in the model (*N_p_*) and the number of residues in the template (*N_t_*):
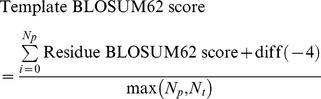
The BLOSUM62 score was the sum of all the Template BLOSUM62 scores normalized by the total number of templates used in the prediction (*N_tot_*):



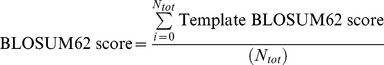
The BLSOUM62 score was then rescaled to lie between 0 and 1, using the maximum residue BLOSUM62 score (MAX_BLO_) and the minimum residue BLOSUM62 score (MIN_BLO_) in the following equation:



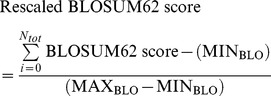
Where MAX_BLO_ = 11 and MIN_BLO_ = −4.

*Equivalent Residue Ligand Distance score:* The Equivalent Residue Ligand Distance score scores the equivalent residues in relation to their distance from the bound ligand. The Equivalent Residue Ligand Distance score was also calculated by firstly making use of an *S*-score. The maximum *S_ik_* score between the equivalent residues in the model and ligand and the *S_jk_* template to the ligand were calculated, where *k* was the closest atom in the ligand to the binding site residues. The differences in distances between the closest residues in the model to the closest atoms in the ligand and the closest residues in the template to the closest atoms in the ligand were calculated (Distance Difference). The Euclidian distance equation was used to calculate the distances in 3D space (x,y,z), between the closest binding site residue to the closest atom in the ligand, using the following equation:
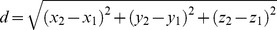
To rescale the score between 0 and 1, the equation Q_SCORE_ =  exp^-Distance Difference^ was used, to convert the Distance Difference. The sum of the Q_SCORE_ for each model-template comparison (Q_TOTAL_) was then divided by the greater value of the number of predicted residues in the model (*N_p_*) and the number of residues in the template (*N_t_*):



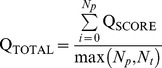
The Equivalent Residue Ligand Distance score was the sum of all the template Q_TOTAL_ scores normalized by the total number of templates used in the prediction (*N_tot_*):



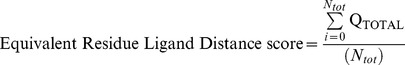



#### Ligand dependent feature scores

In addition to the binding site dependent feature scores, several ligand dependent feature scores were developed, including scores to examine the variation among superposed ligands, in terms of type and category. However, none of the scores were found to correlate well with observed scores and so they were not used in the final FunFOLDQA algorithm. The ligand dependent feature scores are shown for information only.

*Ligand Variation*: This score determines the variation of ligand types in the ligand binding site cluster. The number of each ligand type in the cluster was calculated e.g. if the ligands within the cluster are: ZN-3, FE-2, MN-1, out of a total of 6 ligands in the cluster there are 3 ligand types in the binding site. The total number of ligands in the cluster was also calculated. The Ligand Variation was calculated using the following equation:
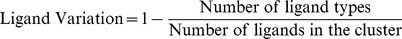



*Ligand Category Variation:* The Ligand Category Variation score is similar to the Ligand Variation score, but focuses on the variation in ligand categories in the ligand binding site cluster. Ligands are classified into 4 categories, based on suggestions by the CASP9 function prediction assessors: metal, DNA/RNA, organic and inorganic. Again, as in the Ligand Variation, the number of ligands in the cluster was calculated. Each ligand within the ligand binding site cluster was categorised into one of the 4 categories. The sum of the different categories present in the ligand binding site cluster was then calculated. The Ligand Category Variation score was subsequently determined using the following equation:
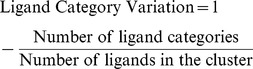



#### Structure dependent feature scores

Several structure dependent feature scores were also developed, which included the mean TM-score [36] of the templates, a score to compare the number of good template superpositions (TM-score ≥ 0.4) to models and the global QA scores from ModFOLDclust2 [37] . The structure dependent feature scores are shown below, only the ModFOLDclust2 score was used in the final FunFOLDQA algorithm.

*Mean TM-score:* The Mean TM-score [36] was calculated to determine the structural relatedness between templates used in the prediction compared to the model. Basically, the mean TM-score was calculated by dividing the sum of TM-scores for all templates used in the prediction by the number of templates in the following equation:
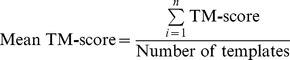



*Template Score:* The Template Score examines the number of templates with biologically relevant ligands with TM-score>0.4 to all templates. Templates need to have a TM-score>0.4, and contain biologically relevant ligands to be used in FunFOLD and FunFOLDQA predictions. The Template Score was calculated using the following equation:
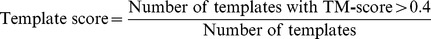



*Model Quality Score:* The Model Quality Score is calculated using ModFOLDclust2 [37], for a detailed description of the ModFOLDlcust2 algorithm see the McGuffin and Roche 2010 paper.

### Determining Which Features to Include in the FunFOLDQA Algorithm

All server models from the CASP8 and CASP9 datasets were downloaded from the CASP website (http://predictioncenter.org/download_area/). The ModFOLDclust2 method [37] was then used to predict the global model quality for server models from both CASP8 and CASP9. Two datasets were produced for each of the CASP8 and CASP9 targets; for example the CASP8 dataset 1 contained the top 10 models for each target, according to ModFOLDclust2, while CASP8 dataset 2 contained 10 models from each target with model quality scores range from good to bad, and not including the top 10 models. Each of the feature scores were initially used to analyse the CASP8 and CASP9 FunFOLD [4] predictions on datasets 1 and datasets 2. Utilizing both datasets, each of the 10 feature scores were compared against both the observed MCC and BDT scores and the Kendall’s *τ*, Spearman’s *ρ* and Pearson’s *r* correlation coefficients were measured. Each of the feature scores were then ranked based on their correlations to the MCC and BDT metrics on the CASP8 and CASP9 datasets 1 and datasets 2.

#### Linear Combination of feature scores (mean score)

Initially, linear combinations of the 5 feature scores with the highest correlations to the MCC and BDT scores (BDTalign, Identity, Rescaled BLOSUM62, Equivalent Residue Ligand Distance and Model Quality) were undertaken. The “Linear Combination” score was compared to both the observed MCC and BDT scores using Kendall’s *τ*, Spearman’s *ρ* and Pearson’s *r* correlation coefficients.

#### Multiple Linear Regression of feature scores

The 5 feature scores with the highest individual correlations were also utilized to carry out multiple linear regression in an attempt to improve the correlation scores. Multiple linear regression was carried out using the R statistics package [38] on the top 5 feature scores, for both CASP8 and CASP9 datasets with the y-value set to either the MCC and BDT metrics to generate weightings for each protein feature. The Multiple Linear Regression score was trained on CASP8 dataset 1 and the subsequent weights were used for testing on CASP9 dataset 1, and vice versa when trained using the CASP9 dataset 1, the weights were used for testing on the CASP8 dataset 1.

#### FunFOLDQA neural network architecture

The 5 feature dependent scores were used as inputs to a feed forward back propagation artificial neural network. The neural network consisted of 3 layers, 5 neurons in the input layer, 5 neurons in the hidden layer and one neuron in the output layer.

To train the FunFOLDQA neural network, the datasets 1 and datasets 2 were combined, producing two new datasets (datasets 3), for both the CASP8 and CASP9 targets. To train the neural network, 5 protein feature scores were used as inputs to the 5 neurons in the input layer and either the MCC or BDT score was the score used for training output. The FunFOLDQA Neural Network was trained on CASP8 dataset 3 and the subsequent weights were used for testing on CASP9 dataset 3, and vice versa when trained using the CASP9 dataset 3, the weights were used for testing on the CASP8 dataset 3. This culminated in the production of 4 sets of results, CASP8 tested on MCC and BDT and CASP9 tested on MCC and BDT.

### Comparison of the Feature Score Combination Methods

Per-target correlations (Kendall’s *τ*, Spearman’s *ρ* and Pearson’s *r*) were calculated to compare each of the methods’ output scores with the observed MCC and BDT scores. The Wilcoxon signed ranked sum test was utilized to compare the per-target correlations, in order to determine whether any method showed a statistically significantly improvement over any of the other methods. Additionally, a receiver operating characteristic analysis was undertaken, using the ROCR [39] plug-in for the R statistical package, with the MCC or BDT score of 0.5 used to determine a boundary between true positive and false positive ligand binding site residue predictions.

**Table 1:** Target-by-target analysis of the correlations for the top single feature score and each combination method (CASP8 data).

Methods	CASP8					
	MCC			BDT		
	Pearson’s *r*	Spearman’s *ρ*	Kendall’s *τ*	Pearson’s *r*	Spearman’s *ρ*	Kendall’s *τ*
**Equivalent Residue Ligand Distance**	0.7517	0.6226	0.4715	0.7509	0.6182	0.4667
**Linear Combination**	0.8086	0.6935	0.5250	**0.7638**	0.6312	**0.4750**
**Multiple Linear Regression**	0.5918	0.5415	0.3917	0.5918	0.5415	0.3917
**Neural Network**	**0.8258**	**0.6982**	**0.5270**	0.6694	**0.6333**	0.4690

Bold values indicate the highest correlation coefficients in each column.

**Table 2:** All versus all Wilcoxon signed ranked sum test analysis, to determine of a significant difference exists between the scoring methods (CASP8 data).

CASP8										
Kendall’s *τ*										
	MCC					BDT				
	Equivalent ResidueLigand Distance		LinearCombination	MultipleLinearRegression	NeuralNetwork	Equivalent ResidueLigand Distance		LinearCombination	MultipleLinearRegression	NeuralNetwork
**Equivalent Residue Ligand Distance**			0.9938	0.7205	0.9958			0.9984	0.1027	0.9693
**Linear Combination**	**0.0068**			**0.0229**	0.0888	**0.0017**			**0.0005**	**0.0455**
**Multiple Linear Regression**	0.2905		0.9791		0.9369	0.9030		0.9995		0.9970
**Neural Network**	**0.0047**		0.9175	0.0682		**0.0329**		0.9582	**0.0032**	
**Spearman’s ** ***ρ***										
	**MCC**					**BDT**				
	**Equivalent Residue** **Ligand Distance**		**Linear** **Combination**	**Multiple** **Linear** **Regression**	**Neural** **Network**	**Equivalent Residue** **Ligand Distance**		**Linear** **Combination**	**Multiple** **Linear** **Regression**	**Neural** **Network**
**Equivalent Residue Ligand Distance**			0.9944	0.6813	0.9891			0.9981	0.0564	0.9671
**Linear Combination**	**0.0062**			**0.0257**	0.0536	**0.0021**			**0.0006**	0.1826
**Multiple Linear Regression**	0.3296		0.9762		0.8348	0.9468		0.9995		0.9971
**Neural Network**	**0.0119**		0.9510	0.1740		**0.0352**		0.8285	**0.0032**	
**Pearson’s ** ***r***										
	**MCC**					**BDT**				
	**Equivalent Residue** **Ligand Distance**	**Linear** **Combination**		**Multiple** **Linear** **Regression**	**Neural** **Network**	**Equivalent Residue** **Ligand Distance**	**Linear** **Combination**		**Multiple** **Linear** **Regression**	**Neural** **Network**
**Equivalent Residue Ligand Distance**		0.9947		0.4832	0.9322		0.9969		0.0742	0.9742
**Linear Combination**	**0.0058**			**0.0069**	**0.0146**	**0.0034**			**0.0028**	0.0705
**Multiple Linear Regression**	0.5279	0.9936			0.9283	0.9295	0.9975			0.9966
**Neural Network**	0.0717	0.9865		0.0758		**0.0275**	0.9331		**0.0037**	

Ho  =  No difference between the methods in the rows and the columns. H1  =  the methods in the row has a higher correlation. Bold values indicate significant p-values (p<0.05).

**Figure 1:**
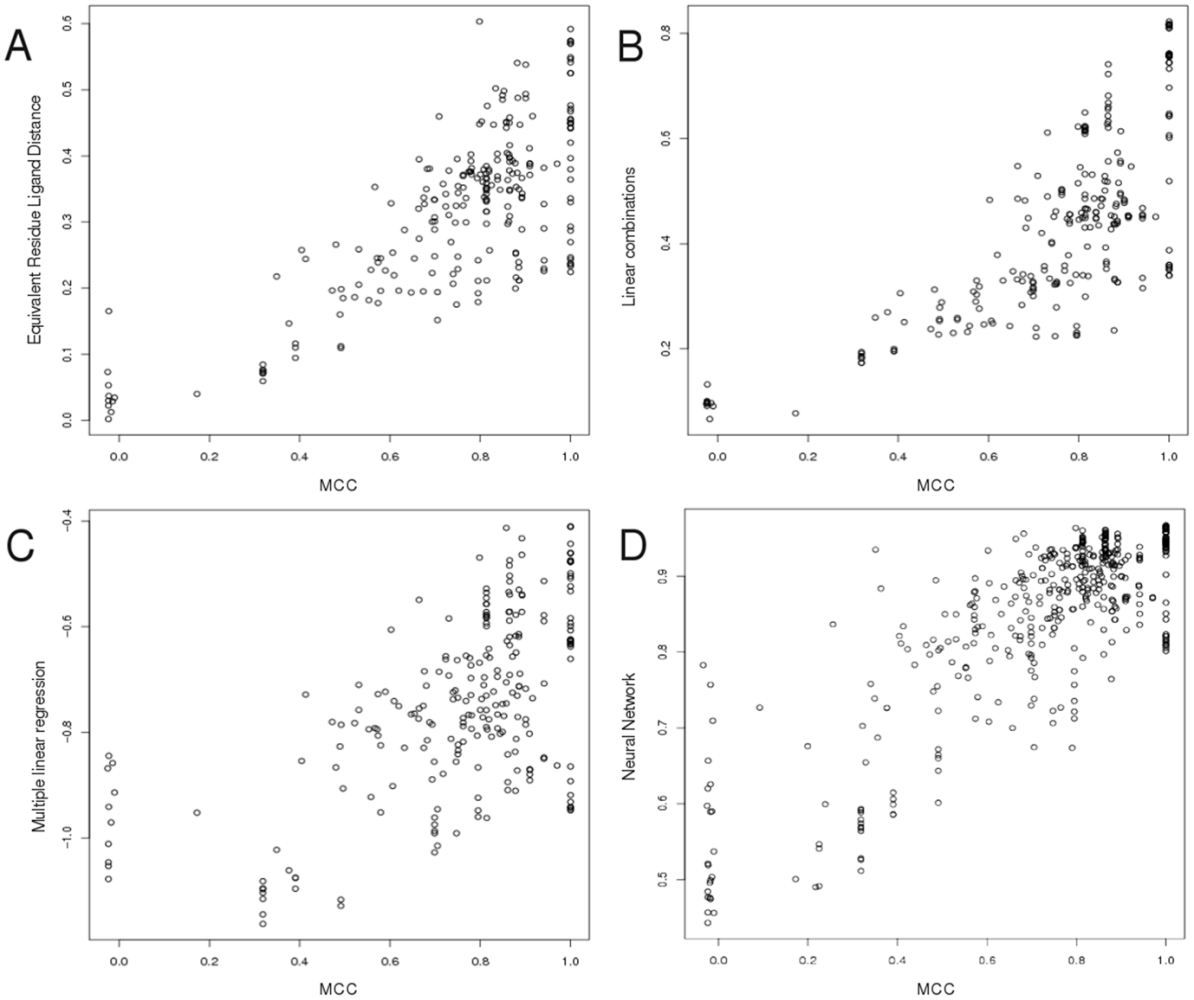
Comparing the top single feature scoring method and each of the combination methods to the observed MCC scores (CASP8 data). A) The Equivalent Residue Ligand Distance score (*ρ* = 0.623). B) The Linear Combination of the 5 feature scores (*ρ* = 0.694). C) The Multiple Linear Regression for the combination of the 5 feature scores (*ρ* = 0.542). D) The 5 feature scores combined using the Neural Network (*ρ* = 0.698).

### Benchmarking of FunFOLDQA Algorithm

The FunFOLDQA Neural Network algorithm was benchmarked using both the CASP8 and CASP9 dataset and both the MCC and BDT metrics. The FunFOLDQA method was benchmarked using only the information concerning templates and models for each target that could be obtained from the CASP8 and CASP9 server predictions. Thus all of the information used was only that which was available to predictors during either CASP prediction season. The FN prediction files for the 27 targets analysed for function prediction in CASP8, the 30 targets analysed for function prediction in CASP9 and all associated 3D server models were downloaded from the CASP website (http://predictioncenter.org/download_area/).

**Table 3:** ROC analysis.

Methods	CASP8						CASP9					
	MCC			BDT			MCC			BDT		
	AUC	SE	AUC_0–0.1_	AUC	SE	AUC_0–0.1_	AUC	SE	AUC_0–0.1_	AUC	SE	AUC_0–0.1_
**Equivalent Residue Ligand Distance**	0.9754	0.0090	0.0792	0.9870	0.0061	0.0889	**0.8333**	0.0251	0.0466	**0.7768**	0.0293	**0.0260**
**Linear Combination**	0.9765	0.0086	0.0883	0.9681	0.0104	0.0846	0.7938	0.0279	0.0398	0.7489	0.0308	0.0172
**Multiple Linear Regression**	0.9089	0.0207	0.0610	0.8974	0.0226	0.0477	0.8048	0.0272	**0.0488**	0.7742	0.0294	0.0256
**Neural Network**	**0.9773**	0.0085	**0.0845**	**0.9903**	0.0052	**0.0904**	0.8003	0.0275	0.0319	0.7634	0.0300	0.0053

SE standard error of AUC [41]; AUC_0–0.1_, AUC for false positive rate between 0 and 10% (false positives were defined as the top function prediction according to each score having an MCC or BDT score > = 0.5). The AUC and AUC_0–0.1_ scores were calculated using ROCR [39]. The highest AUC and AUC_0–0.1_ scores for each CASP prediction session and each performance measure (MCC and BDT) are indicated in bold.

**Figure 2:**
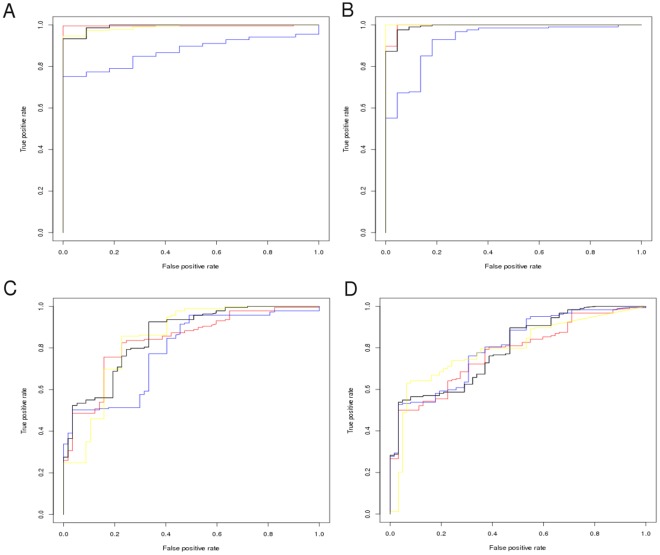
Receiver Operator Characteristic curves for the top single score and each combination method plots. ROC plots for the top single component score (Equivalent Residue Ligand Distances) (black), the Linear Combination (red), Multiple Linear Regression (blue) and Neural Network (yellow) scores for both the MCC and BDT performance metrics on both the CASP8 and CASP9 datasets at a true positive rate of 0.5. A) ROC plot for the MCC performance metric on the CASP8 dataset. B) ROC plot for the BDT performance metric on the CASP8 dataset. C) ROC plot for the MCC performance metric on the CASP9 dataset. D) ROC plot for the BDT performance metric on the CASP9 dataset.

The ModFOLDclust2 method [37] was used to analyze the server models, for each CASP8 and CASP9 target, submitted during both CASP8 and CASP9 prediction seasons. The top 10 models for each target were then used as the starting models for predicting ligand binding residues utilizing FunFOLD [4]. The parent records from each server model were examined in order to construct a list of template PDB IDs for each target, which was available at the time of each CASP prediction season. The list of templates arising from this analysis was subsequently filtered using FASTA [40] to ensure it was 70% non-redundant according to pairwise sequence identity. This type of filtering is in line with that carried out during the construction of the non-redundant fold libraries used by many fold recognition servers, such as IntFOLD-TS [26]. Finally, a maximum of 40 templates were used in our analysis for efficiency. The FunFOLDQA algorithm was subsequently utilized to analyze the FunFOLD binding site residue predictions and produce a quality score for each prediction. The FunFOLDQA score was then used to re-rank the predictions for the top 10 models, from each CASP function prediction target.

The scores for the top ranked FunFOLDQA predictions, for each CASP function prediction target were then compared against all of those from the other function prediction groups participating in CASP8 and CASP9, using the MCC and BDT scores as an indicator of performance. An analysis of the statistical significance between the differences in mean scores was also carried out, similar to that of the official CASP assessments [1], [25]. The Binding-site Distance Test (BDT) score was used with the d_0_ threshold set to 1Å, in order to more stringently assess the accuracy of predictions.

**Figure 3:**
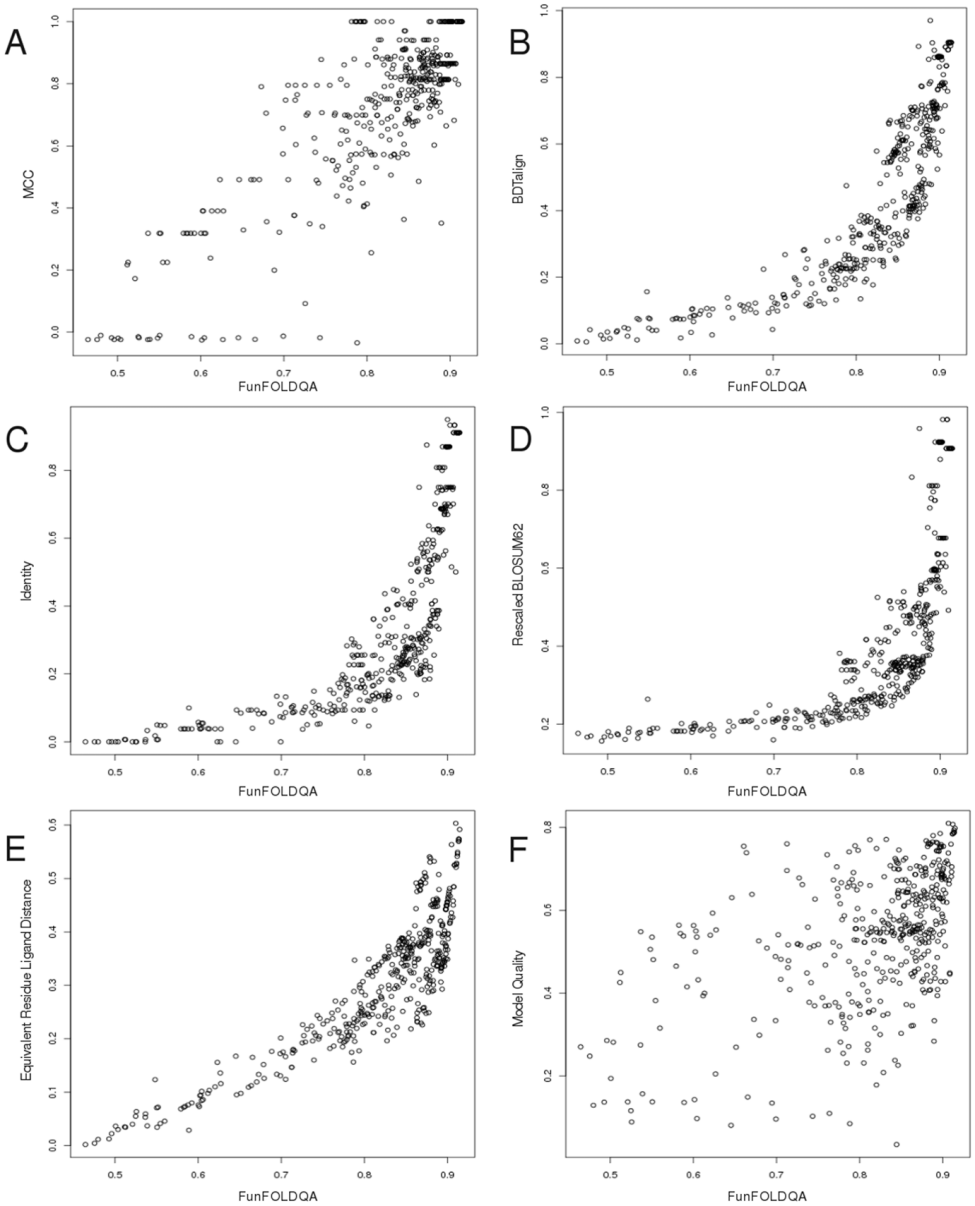
The FunFOLDQA Neural Network scores compared with the observed MCC scores and the feature component scores (CASP8 data). A) The FunFOLDQA Neural Network is plotted against the observed MCC score. B) The BDTalign score. C) The Identity score. D) The Rescaled BLOSUM62 score. E) The Equivalent Residue Ligand Distance score. F) The 3D Model Quality score (ModFOLDclust2).

**Figure 4:**
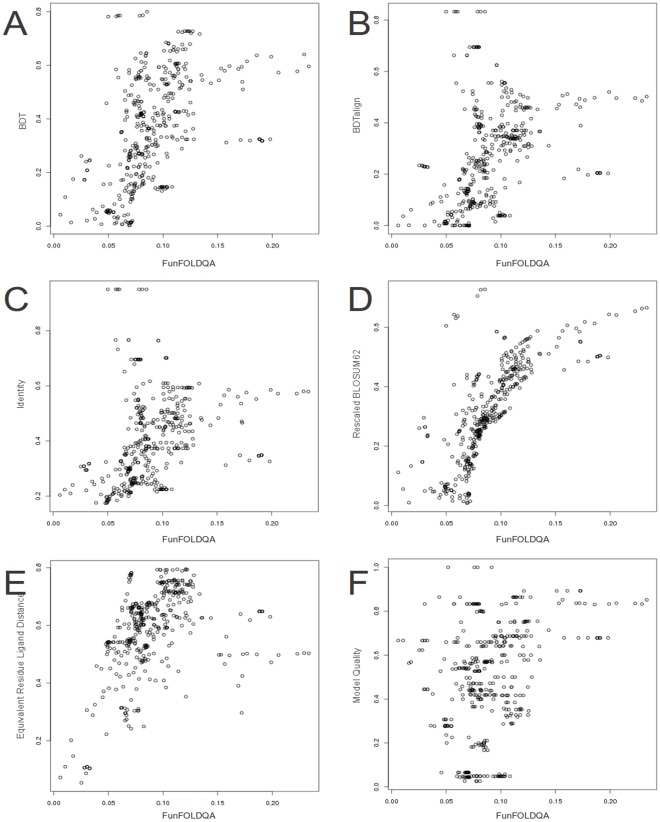
The FunFOLDQA Neural Network scores compared with the observed BDT scores and the feature component scores (CASP8 data). A) The FunFOLDQA Neural Network is plotted against the observed BDT score. B) The BDTalign score. C) The Identity score. D) The Rescaled BLOSUM62 score. E) The Equivalent Residue Ligand Distance score. F) The 3D Model Quality score (ModFOLDclust2).

## Results

Three methods for combination of the 5 FunFOLDQA feature scores: 1. Linear combinations of scores (mean score), 2. Combination of the feature scores using multiple linear regression and 3. Combination using a feed forward neural network with back propagation, are compared to MCC and BDT performance metrics, to determine how closely correlated the predicted feature scores are to observed performance metrics. The Wilcoxon signed ranked sum test is used, to determine if a significant difference in performance exists, between the feed forward neural network and the different combinations of feature scores. Receiver Operating Characteristic (ROC) plots are shown and Area Under the Curve (AUC) and Standard Error (SE) was also calculated, for the combination methods.

The FunFOLDQA algorithm, utilizing the Neural Network for feature score combination, is benchmarked using the set of 27 CASP8 function prediction targets and 30 CASP9 function prediction targets. The performance of FunFOLDQA is compared against that of groups that participated in the CASP8 and CASP9 function prediction categories, along with the FunFOLD method [4].

**Table 4:** All versus all analysis for the top methods in CASP8 along with the FunFOLD and FunFOLDQA methods.

Method	Max	FunFOLDQA	FunFOLD	FN407	FN293	FN202
**Max**		**0.0046**	**0.0001**	**0.0277**	**0.0012**	**0.0003**
**FunFOLDQA**	0.9968		**0.0026**	0.0885	**0.0140**	**0.0004**
**FunFOLD**	0.9999	0.9977		0.4870	0.1280	**0.0010**
**FN407**	0.9750	0.9182	0.5130		**0.0500**	**0.0303**
**FN293**	0.9990	0.9877	0.8720	0.9585		**0.0443**
**FN202**	0.9998	0.9997	0.9999	0.9720	0.9602	

The analysis is based on common subsets of all CASP8 function prediction targets, with a minimum of 10 predictions in common. Predictions are scored using the BDT metric. Ho  =  No difference between the methods in the rows and the columns. H1  =  the methods in the row has a higher correlation. Bold values indicate significant p-values (P<0.05).

**Table 5:** All versus all analysis for the top server methods in CASP9 along with the FunFOLD and FunFOLDQA methods.

Method	FN096	FN339	FN315	FunFOLDQA	FunFOLD	FN236	Max	FN057
**FN096**		**0.0540**	**0.0402**	**0.0476**	**0.0490**	**0.0056**	**0.0069**	**0.0002**
**FN339**	0.9533		0.5000	0.0890	0.1210	**0.0319**	**0.0228**	**0.0014**
**FN315**	0.9624	0.5107		0.1050	0.2280	**0.0252**	**0.0250**	**0.0004**
**FunFOLDQA**	0.9553	0.9156	0.9009		0.6001	0.3216	0.1979	0.0938
**FunFOLD**	0.8790	0.7720	0.6980	0.4115		0.3610	0.1234	0.0590
**FN236**	0.9949	0.9699	0.9765	0.6885	0.6390		0.4277	0.2050
**Max**	0.9937	0.9788	0.9769	0.8828	0.8828	0.5833		0.4459
**FN057**	0.9998	0.9988	0.9996	0.9110	0.9840	0.8035	0.5676	

The analysis is based on common subsets of all CASP9 function prediction targets, with a minimum of 10 predictions in common. Predictions are scored using the BDT metric. Ho  =  No difference between the methods in the rows and the columns. H1  =  the methods in the row has a higher correlation. Bold values indicate significant p-values (p<0.05).

**Figure 5:**
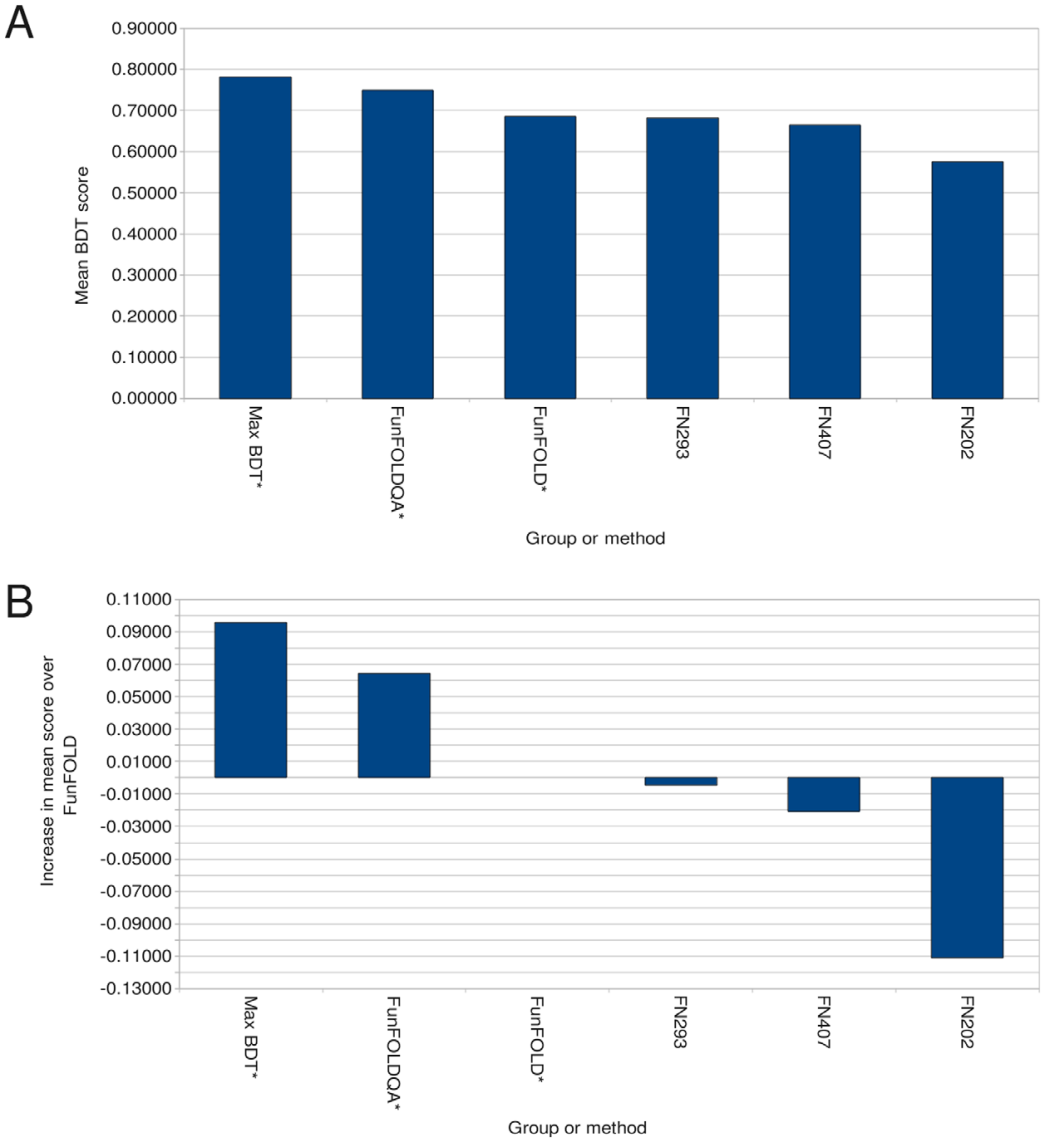
BDT score comparison for the CASP8 benchmarking. A) Mean per-target BDT scores for selected CASP8 function prediction groups along with FunFOLD, FunFOLDQA and the maximum score that could be obtained from FunFOLD. B) The added value, or increase in mean per-target score over FunFOLD (Minimum of 15 predictions) * indicates server method.

**Figure 6:**
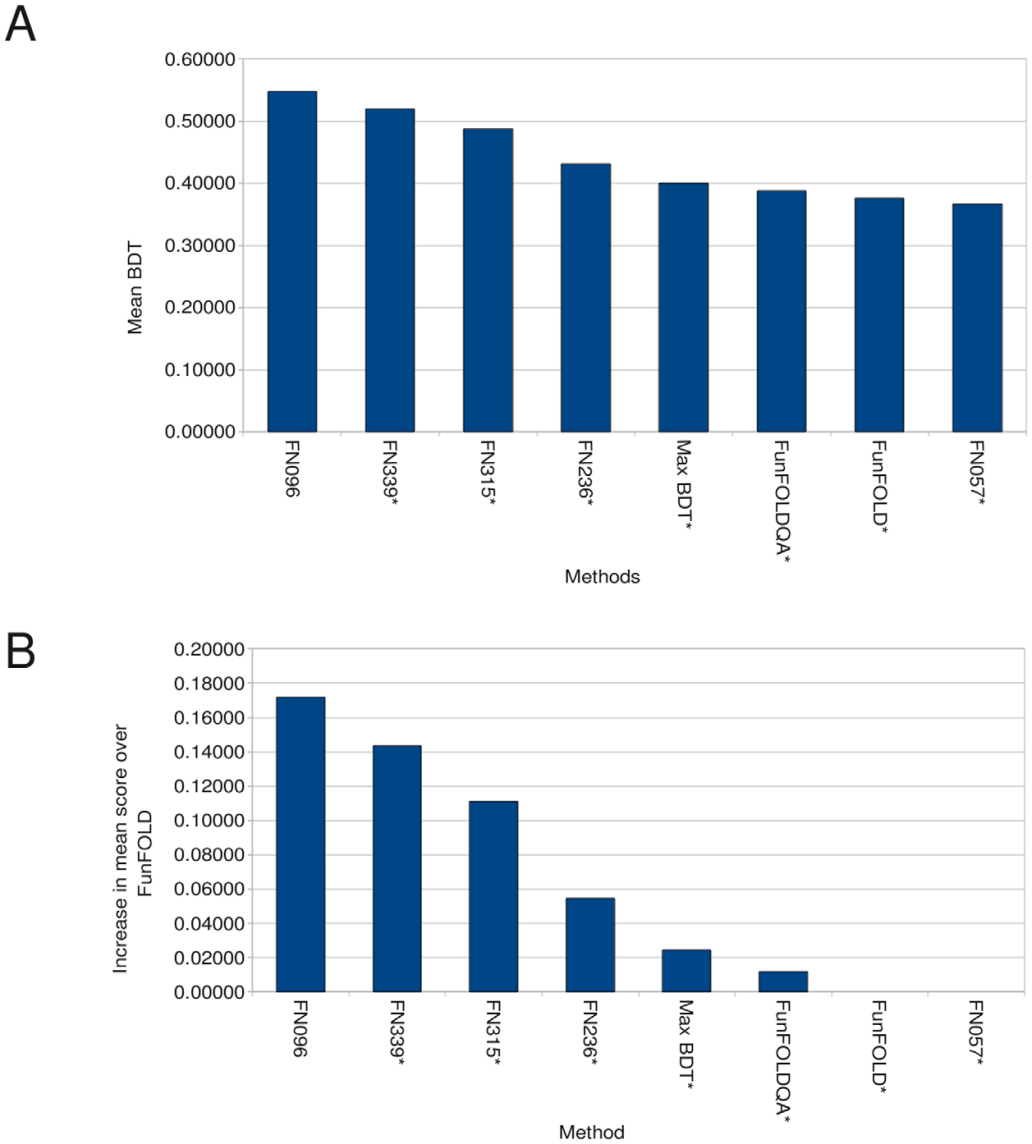
BDT score comparison for the CASP9 benchmarking. A) Mean per-target BDT scores for selected CASP9 function prediction groups along with FunFOLD, FunFOLDQA and the maximum score that could be obtained from FunFOLD. B) The added value, or increase in mean per-target score over FunFOLD (Minimum of 15 predications) * indicates server method.

### Determination of the Best Method for FunFOLDQA Feature Score Combination

When the 10 FunFOLDQA feature dependent scores are initially compared to the MCC and BDT metrics, a large variation in correlations is seen (Kendall’s *τ*, Spearman’s *ρ* and Pearson’s *r* correlation coefficients). It can also be seen that there are several feature scores, which show positive correlation, with the Equivalent Residue Ligand Distance score having the highest correlations to both the MCC and BDT scores over all of the datasets (Figure S1).

On close inspection of the feature dependent score categories, the binding site dependent scores have the highest correlations (*τ*, *ρ* and *r*) to both the MCC and BDT scores. The ligand dependent feature scores are not as closely correlated (*τ*, *ρ* and *r*) to the MCC and BDT metrics. The predictive structure dependent feature scores, are also not well correlated to the observed MCC and BDT scores, but for the CASP8 datasets, interestingly the Model Quality score has a high correlation coefficients, to both the MCC and BDT scores. The 5 feature dependent scores that show the most promise, which were highly correlated to both the MCC and BDT metrics, are the 4 binding site dependent scores (BDTalign, Identity, Rescaled BLOSUM62 and Equivalent Residue Ligand Distance) and one structure dependent feature score (Model Quality) (Figure S1).

**Figure 7:**
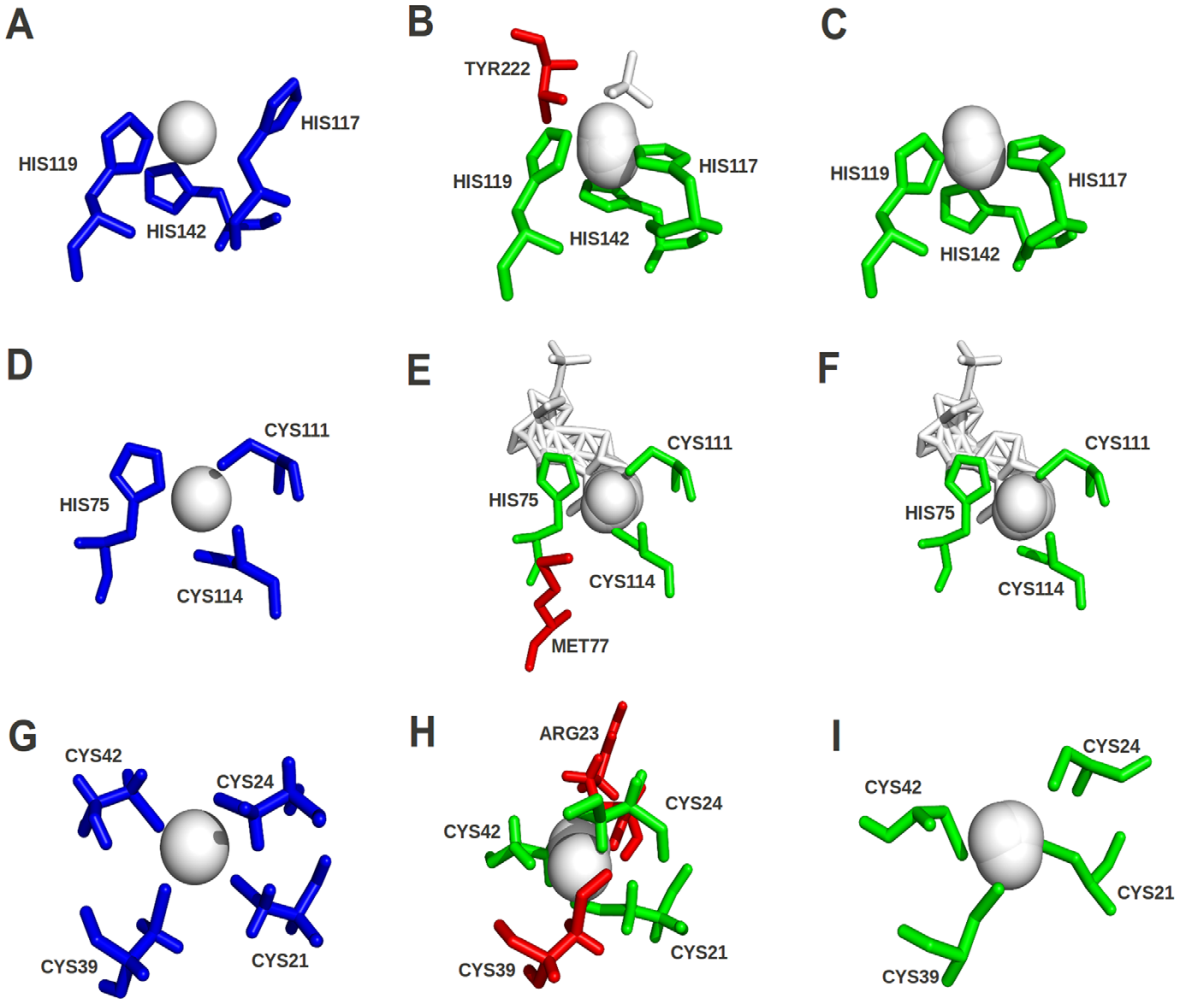
Examples of binding site predictions from CASP8 targets using the FunFOLDQA and FunFOLD methods. The green sticks represent residues in the model that has been correctly predicted as binding to the ligands. The red sticks represent residues that were not predicted or incorrectly predicted as potential ligand binding residues. The blue sticks represent the observed ligand binding site residues in the experimental structure. The white spheres and the white sticks represent ligands either predicted (B, C, E, F, H and I) or observed (A, D and G). A) An example of the observed CASP8 target T0426 (3da2), with the observed binding site residues (117, 119 and 142) and ligands (ZN) shown. B) The predicted binding site from FunFOLD for T0426 with the predicted binding site residues (117, 119, 142 and 222) and ligands (ZN-19 and SO_4_-1) shown. C) An example where FunFOLDQA produces a perfect prediction for CASP8 target T0426 (3da2), with the predicted binding site residues (117, 119 and 142) and ligands (ZN-19 and SO_4_-1) shown. D) An example of the observed CASP8 target T0461 (3dh1), with the observed binding site residues (75, 111 and 114) and ligands shown (ZN). E) The predicted binding site from FunFOLD for T0461 with the predicted binding site residues (75, 77, 111 and 114) and ligands (ZN-17, IMD-1, DDN-1, PO_4_-1 and THU-1) shown. F) An example where FunFOLDQA produces a perfect prediction for CASP8 target T0461 (3dh1), with the predicted binding site residues (75, 111 and 114) and ligands (ZN-17, IMD-1, DDN-1, PO_4_-1 and THU-1) shown. G) An example of the observed CASP8 target T0480 (2k4k), with the observed binding site residues (21, 24, 39 and 42) and ligands (ZN) shown. H) The predicted binding site from FunFOLD for T0480 with the predicted binding site residues (21, 23, 24 and 42) and ligands (ZN-2) shown. I) An example where FunFOLDQA produces a perfect prediction for CASP8 target T0480 (2k4k), with the predicted binding site residues (21, 24, 39 and 42) and ligands (ZN-3) shown.

### Combining the Feature Dependent Scores

When the combined scoring methods and Equivalent Residue Ligand Distance scores are analysed on the CASP8 dataset, the Linear Combination score, shows an improvement in the Kendall’s *τ*, Spearman’s *ρ* and Pearson’s *r*, for correlations to both the MCC and BDT scores, over the best individual feature scores (Table 1), which is shown to be statistically significant in Table 2. The Multiple Linear Regression score results in a decreased correlation to observed MCC and BDT scores, when compared to the Linear Combination method (Table 1), and this was shown to be statistically significant (Table 2). In Table 1 the FunFOLDQA Neural Network score, has overall the highest correlations (Kendall’s *τ*, Spearman’s *ρ* and Pearson’s *r* correlation coefficients), to observed MCC and BDT scores. In Table 2 it can be seen that the Neural Network score is statistically significantly better than the Equivalent Residue Ligand Distance score (*τ, ρ*) for the observed MCC score and shows a statistically significant improvement over both the Equivalent Residue Ligand Distance score and the Multiple Linear Regression score for the observed BDT metric. In Figure 1 plots of the predicted scores to the observed scores for the CASP8 MCC data can be seen.

The Equivalent Residue Ligand Distance score, has a higher correlation (*τ* and *ρ*) than the Neural Network output score for CASP9 data, when compared to the MCC metric (Table S1), but this is not statistically significant (Table S2). For the CASP9 dataset, when the predictive Neural Network score is compared to the observed BDT metric, the correlation coefficient is marginally increased over all other methods (Table S1), however no statistical significance could be measured in this case (Table S2). The Linear Combination score shows a statistically significant improvement over the Equivalent Residue Ligand Distance score and the Multiple Linear Regression score for the CASP9 BDT data (Table S2).

### Receiver Operator Characteristic Analysis

The performance of the feature score combination methods and the top single score (Equivalent Residue Ligand Distance score), for the prediction of the MCC and BDT scores, is also compared using standard ROC analysis. The results in Table 3 show the overall AUC scores for each method, along with the standard error [41], and the AUC scores at low false positive rate. The ROC curves are shown in Figure 2 for both CASP8 and CASP9 MCC and BDT scores.

From the ROC analysis (Table 3 and Figure 2), on the CASP8 data it can be seen that the Neural Network outperforms all of the other methods and this is significant compared with the Multiple Linear Regression score. This shows again the added value of the utilization of a Neural Network for score combination. For the CASP9 data, the single score – Residue Ligand Distance score – outperforms all of the other methods including the Neural Network, but this is not significant when the SE is considered. Unfortunately there is no formal method, which can be used to assess the statistical significance of the observed differences in the AUC scores, however the standard error (SE) score allows us to express the separation between the methods.

### Results from Training the Neural Network

Each feature score was compared to the FunFOLDQA Neural Network output score in an attempt to determine the most important inputs for the Neural Network training (Figures 3, 4, S2 and S3).

For example in Figure 3 for CASP8 data trained on the MCC score, it can be seen that the BDTalign score has the biggest influence on learning (*τ = *0.773, *ρ = *0.926, *r = *0.812), followed by Identity (*τ = *0.763, *ρ = *0.917, *r = *0.714), Rescaled BLOSUM62 (*τ = *0.756, *ρ = *0.915, *r = *0.657), Equivalent Residue Ligand Distance (*τ = *0.688, *ρ = *0.852, *r = *0.868) with the Model Quality score having the least influence on the learning (*τ = *0.361 *ρ = *0.514, *r = *0.516). Interestingly, in Figure 4 for the CASP8 BDT dataset, the Rescaled BLOSUM62 score has the most influence on learning (*τ = *0.884, *ρ = *0.978, *r = *0.950) with the Model Quality score having the lowest correlation (*τ = *0.268, *ρ = *0.379, *r = *0.382). For CASP9 data trained on the MCC score (Figure S2), the Identity score has the highest correlations (*τ = *0.925 *ρ = *0.991, *r = *0.992), again Model Quality plays little influence in the learning of the Neural Network (*τ = *0.037, *ρ = *0.065, *r = *0.088). In Figure S3 for the Neural Network trained on the CASP8 BDT score, when tested on the CASP9 data, the feature dependent scores have the following correlations; BLOSUM62 (*τ = *0.704, *ρ = *0.877, *r = *0.822), Identity (*τ = *0.458, *ρ = *0.641, *r = *0.527) and BDTalign (*τ = *0.439, *ρ = *0.625, *r = *0.550) have the highest correlations, followed by Model Quality (*τ = *0.3305, *ρ = *0.481, *r = *0.512) and the lowest correlation is for the Equivalent Residue Ligand Distance (*τ = *0.112, *ρ = *0.173, *r = *0.011), which does not have as big an influence on learning of the Neural Network.

### Can FunFOLDQA be Used to Add Value to Binding Site Residue Predictions?

The results of an assessment of binding site predictions, similar to the official CASP8 function prediction assessment carried out by Lopez et al. [1], the official CASP9 assessment [25] and our assessment of the FunFOLD method [4], are shown in Tables 4 and 5 and in Figures 5 and 6. The Binding-site Distance Test (BDT) metric is used to measure prediction success; the resulting scores achieved by the different groups and FunFOLD are compared with those from the FunFOLDQA method (The FunFOLDQA method refers to the Neural Network method for feature score combination). The FunFOLDQA method is shown to outperform all other methods tested at CASP8 and the original FunFOLD method [4] according to the mean per-target BDT score (Figure 5).

In Figure 5, the difference in mean BDT performance can be seen. The FunFOLDQA method is 6.43% better than the original FunFOLD method, when tested on the CASP8 function prediction dataset. In addition, the FunFOLDQA method shows>22% improvement over the next best server group FN202’s CASP8 predictions, >9% improvement over group FN293’s CASP8 predictions and a ∼6% improvement over FN407’s CASP8 predictions. The improvement is statistically significant for all CASP8 groups tested and the FunFOLD method, except the manual method by the Lee group FN407 (Table 4). A significant improvement is seen after the addition of quality assessment to the ligand binding site residue prediction method. In our previous study, the original FunFOLD method was not statistically significantly better than group FN293, however using the FunFOLDQA method we can now demonstrate a statistically significant improvement. The maximum BDT score, which can be obtained from the top 10 models using the FunFOLD method, is shown for comparison.

The FunFOLDQA method is also shown to be competitive with the methods tested at CASP9 (Table 5 and Figure 6). The FunFOLDQA method showed no significant difference compared with the FunFOLD method and the top server methods according to mean per-target BDT scores (Table 5) (Partial binding site definitions were used here [25]). According to the Wilcoxon signed ranked sum test, the per-target BDT score for the top manual method (FN096) is statistically significantly better than the FunFOLDQA method, however, no significant difference can be observed between the top server method (FN339 – I_TASSER_FUNCTION [21]) and FunFOLDQA (Table 5). The maximum BDT scores that can be obtained from the top 10 models using the FunFOLD method are also shown in Table 5 and Figure 6 for comparison.

### Example Predictions

In Figure 7 the FunFOLDQA method is shown to add value over using the FunFOLD method alone for T0426 (A - C), T0461 (D – F) and T0480 (G – I). Figure 7C represents accurate predictions for the CASP8 target T0426 (PDBID 3da2) with perfect MCC and BDT scores of 1.0. For comparison, the prediction by the original FunFOLD method (Figure 7B) gave MCC = 0.864 and BDT = 0.750. Analysing the prediction for T0426 in more detail (Figure 7C), the FunFOLDQA method correctly predicted the binding site as being a metal binding site, the observed zinc ligands to be in the correct binding pocket and all correctly predicted binding site residues. However, the FunFOLD method, over predicted one residue for this target – THR222 (Figure 7B - shown in red). The FunFOLDQA method selected a better top prediction where the ligands are superposed in a tighter cluster than the top FunFOLD prediction, thus fewer ligands are closer to residue THR222 and it is not over predicted.

Figure 7F represents an accurate binding site residue prediction for CASP8 target T0461 (PDBID 3dh1), again with perfect MCC and BDT scores. For comparison the original FunFOLD method (Figure 7E) achieved MCC = 0.863 and BDT = 0.75. When the prediction for T0461 (Figure 7F) is analysed in more detail, FunFOLDQA correctly predicts the binding site location, the correct ligand (ZN) and the correct binding site residues. However utilizing the FunFOLD method alone (Figure 7E), again results in an over prediction of one residue MET77 (shown in red).

Another example of the improvement in predictive quality by the addition of FunFOLDQA to the FunFOLD method is seen in Figure 7, for CASP8 target T0480 (PDBID 2k4x) also with perfect MCC and BDT scores. By comparison, the prediction by the original FunFOLD method (Figure 7H) received and MCC score of 0.730 and a BDT score of 0.756. The original FunFOLD method (Figure 7I) over predicts one residue ARG23 (shown in red) and under predicts another residue CYS39 (shown in red).

## Discussion

In this study we describe a novel method, FunFOLDQA, for the quality assessment of ligand binding site residue predictions. The FunFOLDQA algorithm is composed of 5 feature dependent scores. To combine the 5 feature dependent scores 3 methods were tested; simple Linear Combination; Multiple Linear Regression and a Neural Network. The Neural Network showed a statistically significant improvement over both the Linear Combination and the Multiple Linear Regression methods, when the correlations of the predictive output scores to the observed scores (either MCC or BDT) were calculated. ROC analysis was also undertaken, showing that the Neural Network scoring method achieved the largest AUC score and therefore the highest confidence for the CASP8 dataset. We therefore decided to utilize the Neural Network to combine the FunFOLDQA feature dependent scores.

The FunFOLDQA method is a feature based quality assessment method, which assesses the quality of ligand binding site residue predictions, producing an output score between 0 and 1 in relation to the quality of the prediction. A score of 1 indicates a likely perfect prediction and a score close to 0 indicates a likely random prediction. The FunFOLDQA method was initially designed to assess alternative FunFOLD predictions, in an attempt to improve on the predictive quality of the method. We have demonstrated that a statistically significant improvement can be achieved compared with using the FunFOLD algorithm alone. However, the method can also be applied to any other similar method that produces a 3D model and a list of comparable templates as part of its prediction protocol. We provide a downloadable executable of FunFOLDQA, which is usable with any binding site residue prediction tool that is capable of supplying those data as inputs (http://www.reading.ac.uk/bioinf/downloads/).

When designing the FunFOLDQA method, we found it difficult to decide upon which binding site specific feature components to include. Hence, we started with 10 different feature dependent components for the prediction of binding site quality, to initially assess their relationship to the MCC and BDT metrics. The initial scores were derived to quantify features that we found were important in estimating prediction quality during our participation in the CASP9 experiment. The feature dependent scores can be divided into 3 major categories; binding site dependent scores; ligand dependent scores and structure dependent scores.

The binding site dependent feature scores showed positive correlations to both the MCC and BDT metrics. These scores are closely assessing our assumption that structurally similar proteins will have similar binding sites. The structure dependent Model Quality score also showed a positive relationship to both the MCC and BDT metrics. It is assumed that Model Quality is important in binding site predictions, as a bad model, on the whole, should result in a bad binding site prediction and a good model, a good prediction. The other structure dependent scores did not result in a positive relationship to either the MCC or BDT metrics. Presumably this was due to how closely structurally related the templates were. All of the ligand dependent scores showed a weak correlation to the MCC and BDT scores; the correlations (*τ*, *ρ* and *r*) were low and therefore were not utilized in the final score. We postulate that the variation in ligand size, ligand type and chemotype category found across templates does not play a direct role in the prediction.

Initially the 5 feature dependent scores; BDTalign, Identity, Rescaled BLOSUM62, Equivalent Residue Ligand Distance and Model Quality were combined linearly i.e. their mean score was calculated. This was found to improve the correlations to both the MCC and BDT metrics compared to the individual component scores. The ideal score would produce a correlation close to one, and would be a direct replacement for the MCC or BDT metric, when the solved structure data is not available. Therefore we attempted to improve on this score further using multiple linear regression and a neural network. However, when multiple linear regression was used to combine scores, this resulted in a slight decrease in the correlation coefficients when compared to the observed MCC and BDT scores. The decrease in the Kendall’s *τ*, Spearman’s *ρ* and Pearson’s *r* correlation coefficients is due presumably to the lack of linearity of the component scores (Figure 1). We then attempted to train a feed forward neural network with back propagation, using the 5 component scores as neurons in the input layer, 5 neurons in the hidden layer and either the MCC or BDT metrics as the neuron in the output layer and this was found to improve predictions further still (albeit marginally).

Overall for the CASP8 data the Neural Network showed the most improvement over the Multiple Linear Regression method and the Equivalent Residue Ligand Distance score (Table 2). Interestingly for the CASP9 MCC data for Kendall’s *τ*, Spearman’s *ρ* correlation coefficient no significant difference is seen between the combination methods, but for Pearson’s *r* the Neural Network score shows an improvement over Multiple Linear Regression. For the CASP9 BDT data, the Linear Combination score shows a significant improvement over Equivalent Residue Ligand Distances and Multiple Linear Regression scores for the three correlation coefficients, with no significant difference between the Neural Network and the other methods (Table S2). Using the Multiple Linear Regression assumes a linear relationship between the scores, also utilizing the linear combinations assumes that all the scores play an equal role in assessing the ligand binding site residue prediction results. Conversely, the neural network should learn the relationship between the input scores and the observed output scores (MCC or BDT), thus weighting the scores more appropriately.

The ROC analysis provides a useful benchmark for gauging the consistency of the output scores for the combination methods. In order to have a high level of confidence in the binding site quality predictions, the scores must be comparable from one target to the next. The confidence of the output scores for the combination methods can be compared by studying the plots of the true positive rate to false positive rate (Figure 2). For the CASP8 ROC analysis the Neural Network method has the largest AUC score (Table 3), thus the methods produces less false positive hits for every true positive hit, when compared to the other combination methods and the single score. However their difference in performance is only significant over the Multiple Linear Regression method on the CASP8 data. For the CASP9 ROC analysis, the Equivalent Residue Ligand Distance score has the highest AUC score (Table 3). However, there is no significant separation between the methods according to the standard error of the AUC scores. The AUC score reflects the score consistency across targets, which is useful in this assessment. The AUC analysis examines the number of false positive hits i.e. incorrect predictions achieving good predictive scores compared to the number of true positive hits. For the CASP8 data, the Neural Network provides the optimal score combination for producing the best predicted binding site quality. For CASP9, the single score or the Multiple Linear Regression method are sufficient. These results reflect the correlation results shown in Table 1 and Figure 1.

When FunFOLDQA is used in combination with the FunFOLD [4] ligand binding site prediction tool, a significant improvement over all of the server methods that were tested at CASP8 is seen, as well as one of the top manual groups (FN293) and FunFOLD. This shows that the addition of FunFOLDQA, improves the predictive value of the FunFOLD algorithm. As with the original FunFOLD method, FunFOLDQA was found to be competitive with all of the top server groups that participated in CASP9 and again shows a marginal improvement.

Although the FunFOLDQA Neural Network based quality assessment method has a good correlation to the MCC and BDT scores with a Spearman’s *ρ* of ∼0.7, there is room for improvement, to increase the correlation to a score closer to 1.0. In addition, even though the FunFOLDQA method results in a 6.39% improvement (CASP8) over the FunFOLD method, it does not always pick the predictions with the top MCC or BDT scores. The maximum BDT score, which can be achieved from the predictions on the top 10 models, would provide a further improvement of ∼2.9% above the FunFOLDQA method. This is the maximum score that could be achieved using the FunFOLD method with the models available. The use of alternative machine learning methods for score combination may improve the output score and bring it closer in line with the MCC and BDT metrics. The addition of some new feature dependent scores may also help to improve the method; these may include a better score to assess the ligand variation and look at the physiochemical properties of the binding sites residues in the model compared to the template.

### Conclusions

The FunFOLDQA score provides an accurate measure of binding site prediction quality that reflects the MCC or BDT metrics, prior to the availability of structural data. The FunFOLDQA Neural Network helps to reduce the number of false positive predictions and has a strong correlation to both the MCC and BDT metrics. The ability to predict the quality of a binding site residue prediction is important for the experimentalist who wishes to know how reliable the prediction might be and whether the prediction should be used to inform future experiments. This type of score is directly in line with the CASP9 assessor suggestion, that predictors also provide scores that assess the quality of their function predictions [25]. Furthermore from a predictor’s perspective, the FunFOLDQA score is also shown to add significant value to ligand binding site prediction, for example resulting in a 6.39% improvement over our previous FunFOLD method.

## Supporting Information

Figure S1 **Comparing the single feature scoring methods to the observed MCC scores (CASP8 data).** A) The BDTalign score (*ρ* = 0.665). B) The Identity score (*ρ* = 0.677). C) The Rescaled BLOSUM62 score (*ρ* = 0.733). D) The Equivalent Residue Ligand Distance score (*ρ* = 0.623). E) The Ligand Volume Variation score (*ρ* = −0.358). F) The Ligand Variation score (*ρ* = 0.101). G) Ligand Category score (*ρ* = −0.002). H) The Mean TM-score (*ρ* = −0.044). I) the Template score (*ρ* = 0.175). J) The Model Quality score (*ρ* = 0.411).
(TIF)

Figure S2 **The FunFOLDQA neural network scores compared with the observed MCC scores and the feature component scores (CASP9 data).** A) The FunFOLDQA neural network is plotted against the observed MCC score. B) The BDTalign score. C) The Identity score. D) The Rescaled BLOSUM62 score. E) The Equivalent Residue Ligand Distance score. F) The 3D model quality score (ModFOLDclust2).
(TIF)

Figure S3 **The FunFOLDQA neural network scores compared with the observed BDT scores and the feature component scores (CASP9 data).** A) The FunFOLDQA neural network is plotted against the observed BDT score. B) The BDTalign score. C) The Identity score. D) The Rescaled BLOSUM62 score. E) The Equivalent Residue Ligand Distance score. F) The 3D model quality score (ModFOLDclust2).
(TIF)

Table S1 **Target-by-target analysis of the correlations for the top single feature score and each combination method (CASP9 data).** Bold values indicate the highest correlation coefficients in each column.
(DOC)

Table S2 **All versus all Wilcoxon signed ranked sum test analysis, to determine if a significant difference exists between the scoring methods (CASP9 data).** Ho = No difference between the methods in the rows and the columns. H1 = the methods in the row has a higher correlation. Bold values indicate significant p-values (p < 0.05).
(DOC)
