# Supplementary figures and images for: FunFOLDQA: A Quality Assessment Tool for Protein-Ligand Binding Site Residue Predictions
Source: PLoS One. 2012 May 30;7(5):e38219. doi: 10.1371/journal.pone.0038219 (PMC3364224; doi:10.1371/journal.pone.0038219)

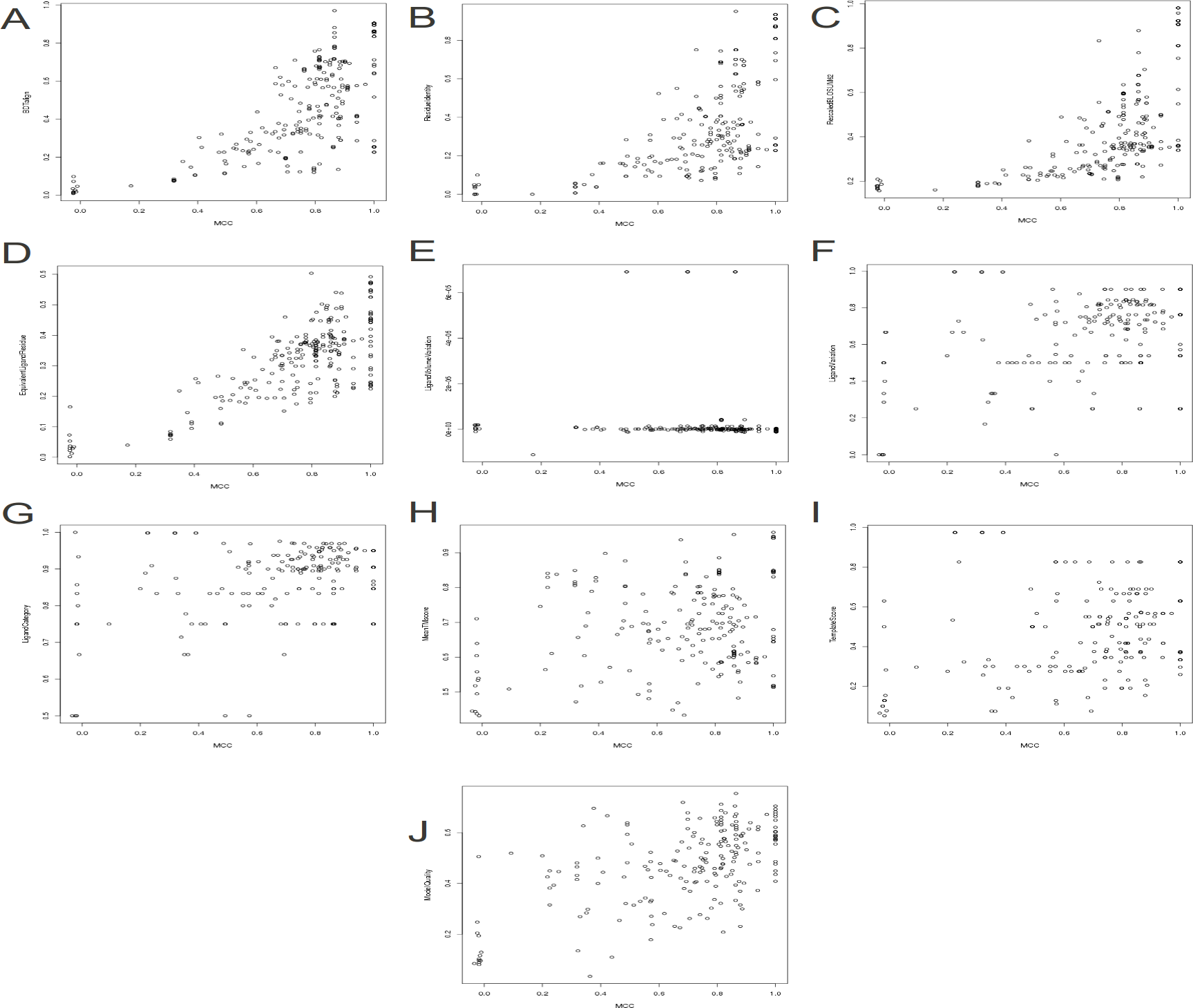

Supplement: Figure S1 — Comparing the single feature scoring methods to the observed MCC scores (CASP8 data). A) The BDTalign score (ρ = 0.665). B) The Identity score (ρ = 0.677). C) The Rescaled BLOSUM62 score (ρ = 0.733). D) The Equivalent Residue Ligand Distance score (ρ = 0.623). E) The Ligand Volume Variation score (ρ = −0.358). F) The Ligand Variation score (ρ = 0.101). G) Ligand Category score (ρ = −0.002). H) The Mean TM-score (ρ = −0.044). I) the Template score (ρ = 0.175). J) The Model Quality score (ρ = 0.411). (TIF) [file pone.0038219.s001.tif]

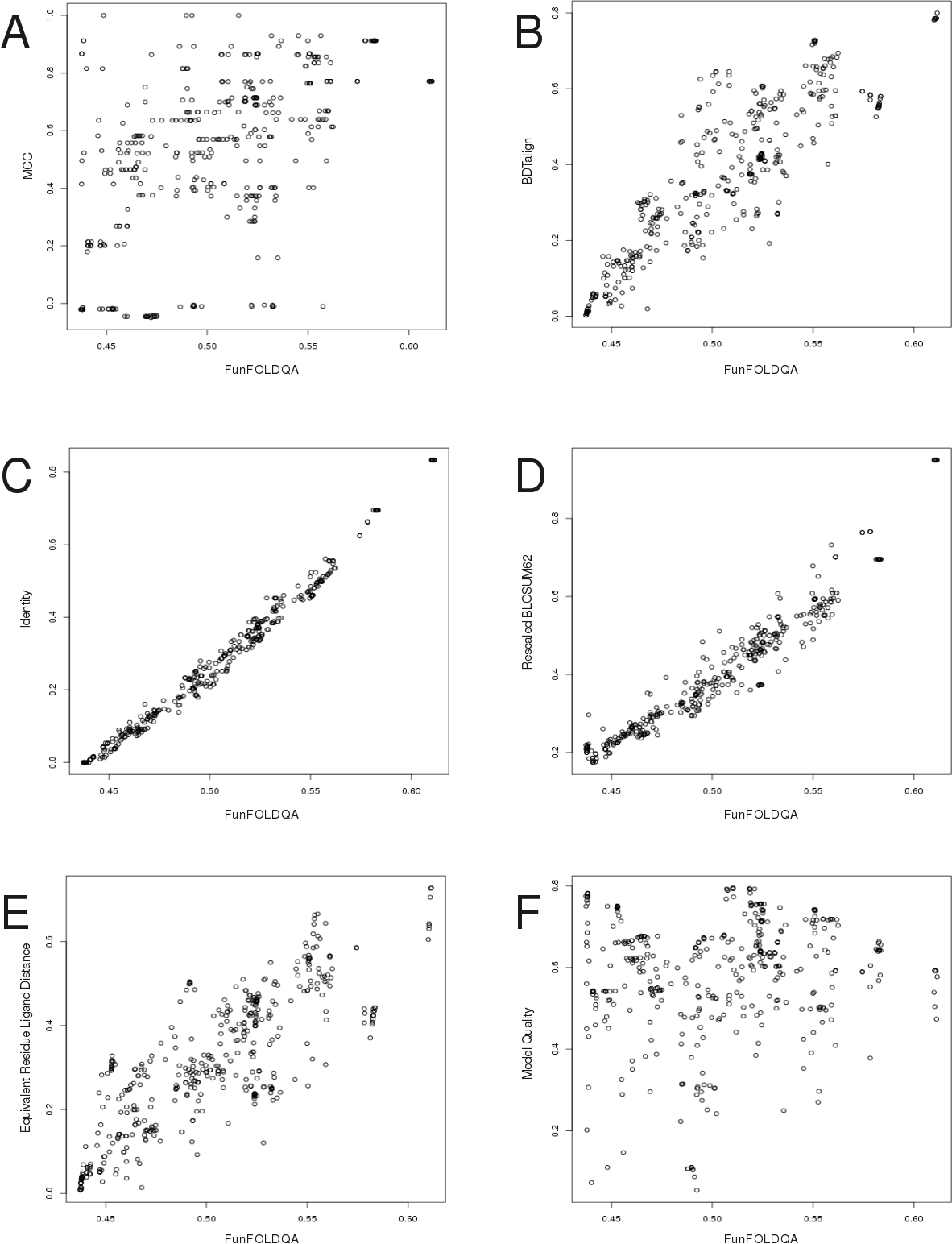

Supplement: Figure S2 — The FunFOLDQA neural network scores compared with the observed MCC scores and the feature component scores (CASP9 data). A) The FunFOLDQA neural network is plotted against the observed MCC score. B) The BDTalign score. C) The Identity score. D) The Rescaled BLOSUM62 score. E) The Equivalent Residue Ligand Distance score. F) The 3D model quality score (ModFOLDclust2). (TIF) [file pone.0038219.s002.tif]

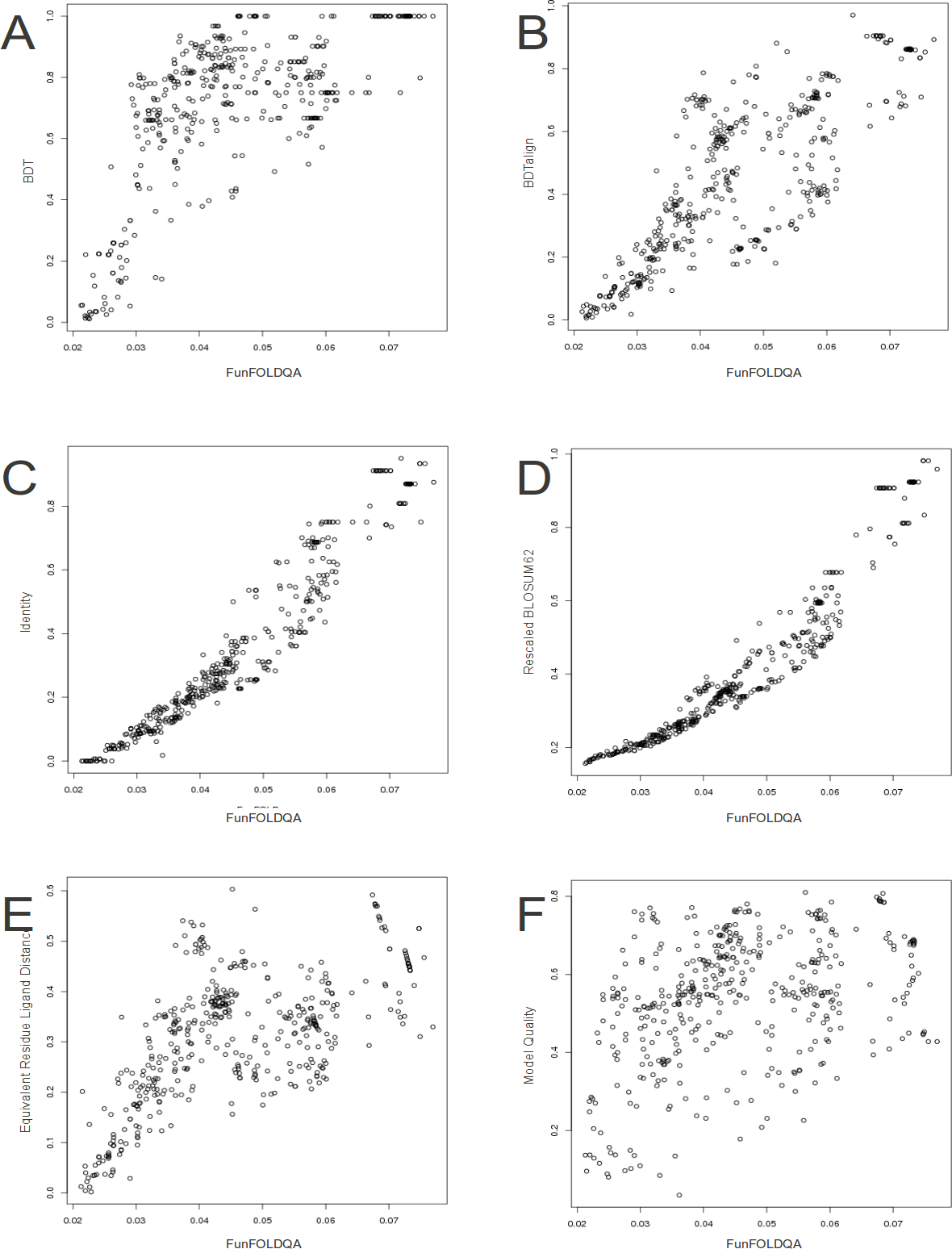

Supplement: Figure S3 — The FunFOLDQA neural network scores compared with the observed BDT scores and the feature component scores (CASP9 data). A) The FunFOLDQA neural network is plotted against the observed BDT score. B) The BDTalign score. C) The Identity score. D) The Rescaled BLOSUM62 score. E) The Equivalent Residue Ligand Distance score. F) The 3D model quality score (ModFOLDclust2). (TIF) [file pone.0038219.s003.tif]
