# Supplementary material for: FunFOLDQA: A Quality Assessment Tool for Protein-Ligand Binding Site Residue Predictions
Source: PLoS One. 2012 May 30;7(5):e38219. doi: 10.1371/journal.pone.0038219 (PMC3364224; doi:10.1371/journal.pone.0038219)
Supplement: Table S1 — Target-by-target analysis of the correlations for the top single feature score and each combination method (CASP9 data). Bold values indicate the highest correlation coefficients in each column. (DOC) [file pone.0038219.s004.doc]

**Table S1. Target-by-target analysis of the correlations for the top single feature score and each combination method (CASP9 data)**. Bold values indicate the highest correlation coefficients in each column.

| **Methods** | **CASP9** | | | | | |
| --- | --- | --- | --- | --- | --- | --- |
|  | **MCC** | | | **BDT** | | |
|  | **Pearson’s *r*** | **Spearman’s *ρ*** | **Kendall’s *τ*** | **Pearson’s *r*** | **Spearman’s *ρ*** | **Kendall’s *τ*** |
| **Equivalent Residue Ligand Distance** | **0.6228** | **0.5995** | 0.4263 | 0.6010 | 0.5871 | 0.4156 |
| **Linear Combination** | 0.5597 | 0.5891 | **0.4272** | 0.5003 | 0.5097 | 0.3511 |
| **Multiple Linear Regression** | 0.5406 | 0.5846 | 0.4206 | 0.5849 | 0.5781 | 0.4028 |
| **Neural Network** | 0.5667 | 0.5753 | 0.4212 | **0.7028** | **0.7723** | **0.5800** |
