# Supplementary material for: FunFOLDQA: A Quality Assessment Tool for Protein-Ligand Binding Site Residue Predictions
Source: PLoS One. 2012 May 30;7(5):e38219. doi: 10.1371/journal.pone.0038219 (PMC3364224; doi:10.1371/journal.pone.0038219)
Supplement: Table S2 — All versus all Wilcoxon signed ranked sum test analysis, to determine if a significant difference exists between the scoring methods (CASP9 data). Ho = No difference between the methods in the rows and the columns. H1 = the methods in the row has a higher correlation. Bold values indicate significant p-values (p < 0.05). (DOC) [file pone.0038219.s005.doc]

**Table S2. All versus all Wilcoxon signed ranked sum test analysis, to determine if a significant difference exists between the scoring methods (CASP9 data)**. Ho = No difference between the methods in the rows and the columns. H1 = the methods in the row has a higher correlation. Bold values indicate significant p-values (p < 0.05).

| **Methods** | **CASP9** | | | | | | | |
| --- | --- | --- | --- | --- | --- | --- | --- | --- |
|  | Kendall’s *τ* | | | | | | | |
|  | **MCC** | | | | **BDT** | | | |
|  | **Equivalent Residue Ligand Distance** | **Linear Combination** | **Multiple Linear Regression** | **Neural Network** | **Equivalent Residue Ligand Distance** | **Linear Combination** | **Multiple Linear Regression** | **Neural Network** |
| **Equivalent Residue Ligand Distance** |  | 0.1818 | 0.2072 | 0.4440 |  | 0.9981 | 0.6698 | 0.8511 |
| **Linear Combination** | 0.8360 |  | 0.6118 | 0.8957 | **0.0023** |  | **0.0065** | 0.2047 |
| **Multiple Linear Regression** | 0.8102 | 0.4064 |  | 0.6197 | 0.3532 | 0.9942 |  | 0.7770 |
| **Neural Network** | 0.5718 | 0.1174 | 0.3969 |  | 0.1601 | 0.8065 | 0.2361 |  |
| Spearman’s *ρ* | | | | | | | | |
|  | **MCC** | | | | **BDT** | | | |
|  | **Equivalent Residue Ligand Distance** | **Linear Combination** | **Multiple Linear Regression** | **Neural Network** | **Equivalent Residue Ligand Distance** | **Linear Combination** | **Multiple Linear Regression** | **Neural Network** |
| **Equivalent Residue Ligand Distance** |  | 0.0817 | 0.1601 | 0.3074 |  | 0.9971 | 0.7551 | 0.8743 |
| **Linear Combination** | 0.9247 |  | 0.6650 | 0.8974 | **0.0033** |  | **0.0065** | 0.2283 |
| **Multiple Linear Regression** | 0.8534 | 0.3524 |  | 0.5667 | 0.2651 | 0.9942 |  | 0.6338 |
| **Neural Network** | 0.7065 | 0.1121 | 0.4480 |  | 0.1342 | 0.7837 | 0.3814 |  |
| Pearson’s *r* | | | | | | | | |
|  | **MCC** | | | | **BDT** | | | |
|  | **Equivalent Residue Ligand Distance** | **Linear Combination** | **Multiple Linear Regression** | **Neural Network** | **Equivalent Residue Ligand Distance** | **Linear Combination** | **Multiple Linear Regression** | **Neural Network** |
| **Equivalent Residue Ligand Distance** |  | 0.8398 | 0.4191 | 0.9360 |  | 0.9589 | 0.0640 | 0.8855 |
| **Linear Combination** | 0.1688 |  | 0.0522 | 0.9484 | **0.0444** |  | **0.0028** | 0.5407 |
| **Multiple Linear Regression** | 0.5941 | 0.9517 |  | 0.9825 | 0.9403 | 0.9976 |  | 0.9105 |
| **Neural Network** | 0.0686 | 0.0555 | **0.0192** |  | 0.1214 | 0.4729 | 0.0953 |  |
